# Supplementary material for: The Advertising Policies of Major Social Media Platforms Overlook the Imperative to Restrict the Exposure of Children and Adolescents to the Promotion of Unhealthy Foods and Beverages
Source: Int J Environ Res Public Health. 2020 Jun 11;17(11):4172. doi: 10.3390/ijerph17114172 (PMC7312784; doi:10.3390/ijerph17114172)
Supplement: Supplementary file 1 [file ijerph-17-04172-s001.pdf]

Title: The advertising policies of major social media platforms overlook the imperative to restrict the exposure of children and adolescents to the promotion of unhealthy foods and beverages

Supplementary material

Table S1: Advertising policies of sixteen major social media platforms in relation to unhealthy food, tobacco, alcohol, gambling and weight-loss, as at December 2019. Refer to

Table S2 for links to advertising policy documents

**Platform**	**Advertising provisions as at December 2019**										
	**Unhealthy foods**		**Tobacco**		**Alcohol**		**Gambling**		**Weight loss**		**Other policies related to content targeted at minors**
	General	***Minors***	***General***	***Minors***	***General***	***Minors***	***General***	***Minors***	***General***	***Minors***	
Facebook	**NO**	**NO**	**YES - Prohibited Content**<br><br>- Adverts **must not promote the sale or use of tobacco products** and related paraphernalia		**YES - Restricted Content**<br><br>**-** Ads that **promote or reference alcohol must comply with all applicable local laws, required or established industry codes, guidelines, licences and approvals, and include age and country targeting criteria** consistent with Facebook's targeting guidelines and applicable local laws.<br><br>- Note that ads promoting or referencing alcohol are prohibited in some countries, including, but not limited to: Afghanistan, Brunei, Bangladesh, Egypt, Gambia, Kuwait, Libya, Lithuania,	**YES - Restricted Content**<br><br>***Targeting requirements:*** If you choose to run adverts that promote or reference alcohol, you must make sure that you follow local laws and target your adverts appropriately, including targeting your adverts to people aged 18 years or older in all permitted countries, except the following:<br><br>***India:***<br>- 25 years or older in Chandigarh, Delhi, Haryana, Maharashtra and Punjab.<br>- 21 years or older in Andhra Pradesh, Arunachal Pradesh, Assam, Chhattisgarh, Dadra and	**YES - Restricted Content**<br><br>- Adverts that **promote or facilitate online real money gambling, real money games of skill or real money lotteries, including online real money casinos, sport books, bingo or poker, are only allowed with prior written permission**.<br>- Authorised gambling, games of skill or lottery adverts **must target people over the age of 18 who are in jurisdictions for which permission has been granted**	**YES - Restricted Content**<br><br>- Authorised gambling, games of skill or lottery adverts **must target people over the age of 18 who are in jurisdictions for which permission has been granted**	**YES - Prohibited Content**<br><br>**-** Ads must not contain "before-and-after" images or images that contain unexpected or unlikely results. Ad content must not imply or attempt to generate negative self-perception in order to promote diet, weight loss or other health-related products.	**YES - Restricted Content**<br><br>- Ads marketed at or for weight loss must be targeted to people aged 18 years or older.	

Platform	Advertising provisions as at December 2019										
	Unhealthy foods		Tobacco		Alcohol		Gambling		Weight loss		Other policies related to content targeted at minors
	General	*Minors*	*General*	*Minors*	*General*	*Minors*	*General*	*Minors*	*General*	*Minors*	
					Norway, Pakistan, Russia, Saudi Arabia, Turkey, United Arab Emirates and Yemen	Nagar Haveli, Dama and Diu, Goa, Jammu and Kashmir, Jharkhand, Kerala, Madhya Pradesh, Meghalaya, Odisha, Tamil Nadu, Telengana, Tripura, Uttar Pradesh, Uttarakhand or West Bengal.<br>- 18 years or older in the Andaman and Nicobar Islands, Himachal Pradesh, Karnataka, Mizoram, Puducherry, Rajasthan and Sikkim.<br>- Alcohol is prohibited in Bihar, Gujarat, Lakshadweep, Manipur and Nagaland.<br><br>***Other countries***<br>- 20 years or older in Japan, Iceland, Thailand and Paraguay<br>- 21 years or older in					

Platform	Advertising provisions as at December 2019										
	**Unhealthy foods**		**Tobacco**		**Alcohol**		**Gambling**		**Weight loss**		**Other policies related to content targeted at minors**
	General	*Minors*	*General*	*Minors*	*General*	*Minors*	*General*	*Minors*	*General*	*Minors*	
						Cameroon, Micronesia, Palau, Solomon Islands, Sri Lanka and the US<br>- 19 years or older in Canada, Korea and Nicaragua.<br>- 25 years or older in Sweden					
YouTube	**NO**	**NO**	**YES - Prohibited Content (Dangerous products or services)**<br><br>The following is not allowed:<br>- Ads for tobacco or any products containing tobacco<br>- Ads for products that form a component part of a tobacco product, as well as products and services that directly facilitate or promote tobacco consumption<br>- Ads for products		**YES - Restricted Content**<br><br>**-** We abide by local alcohol laws and industry standards, so we don't allow certain kinds of alcohol-related advertising, both for alcohol and drinks that resemble alcohol.<br>- Some types of alcohol-related ads are allowed if they meet the [stipulated] policies, don't target minors and target only countries that are explicitly allowed to show alcohol ads	**YES - Restricted Content**<br><br>**-** We abide by local alcohol laws and industry standards, so we don't allow certain kinds of alcohol-related advertising, both for alcohol and drinks that resemble alcohol.<br>**- Some types of alcohol-related ads are allowed if** they meet the [stipulated] policies, **don't target minors** and target only countries that are explicitly allowed to show alcohol ads	**YES - Restricted Content**<br><br>- We support responsible gambling advertising and abide by local gambling laws and industry standards, so we don't allow certain kinds of gambling-related advertising.<br>- Gambling-related ads are allowed if they comply with the policies below and the advertiser has received the proper Google Ads certification.<br>- Gambling ads must target approved countries, have a landing page that	**YES - Restricted Content**<br><br>- **Gambling ads must** target approved countries, have a landing page that displays information about responsible gambling and **never target minors**	**YES - Restricted Content (Healthcare and Medicines - Unapproved Substances)**<br><br>- Google doesn't allow the promotion of the following content, irrespective of any claims of legality:<br>Products containing human chorionic gonadotropin (hCG) in relation to weight loss or weight control, or when promoted in conjunction		

Platform	Advertising provisions as at December 2019										
	**Unhealthy foods**		**Tobacco**		**Alcohol**		**Gambling**		**Weight loss**		**Other policies related to content targeted at minors**
	General	***Minors***	***General***	***Minors***	***General***	***Minors***	***General***	***Minors***	***General***	***Minors***	
			designed to simulate tobacco smoking				displays information about responsible gambling and never target minors.		with anabolic steroids		
YouTube Kids (for children under 13)		**YES – Prohibited**<br><br>- Products related to consumable food and drinks are prohibited, regardless of nutrition content.	**YES – Prohibited**<br><br>- Products that are regulated or illegal to advertise to children, including Prohibited Content and Restricted Content are prohibited. This also includes products that may pose safety risks for children.	**YES – Prohibited**<br><br>- Products that are regulated or illegal to advertise to children, including Prohibited Content and Restricted Content are prohibited. This also includes products that may pose safety risks for children.	**YES – Prohibited**<br><br>- Products that are regulated or illegal to advertise to children, including Prohibited Content and Restricted Content are prohibited. This also includes products that may pose safety risks for children.	**YES - Prohibited**<br><br>- Products that are regulated or illegal to advertise to children, including Prohibited Content and Restricted Content are prohibited. This also includes products that may pose safety risks for children.	**YES – Prohibited**<br><br>- Products that are regulated or illegal to advertise to children, including Prohibited Content and Restricted Content are prohibited. This also includes products that may pose safety risks for children.	**YES – Prohibited**<br><br>- Products that are regulated or illegal to advertise to children, including Prohibited Content and Restricted Content are prohibited. This also includes products that may pose safety risks for children.	**YES - Prohibited**<br><br>- Products related to external personal care, fitness, exercise, weight-loss, diet and nutrition are prohibited.	**YES – Prohibited**<br><br>- Products related to external personal care, fitness, exercise, weight-loss, diet and nutrition are prohibited.	**YES - Restricted**<br><br>***- All YouTube Kids Paid Ads must be pre-approved by YouTube's policy team prior to being served in the YouTube Kids app.***<br>- We prohibit interest-based advertising in YouTube Kids.<br>- Incitement to purchase<br>- Promotions or content that incites children to purchase a product or service or to urge parents or others to buy the item.<br>- Misleading and deceptive claims:<br>(i) Paid Ads cannot be misleading to children and

Platform	Advertising provisions as at December 2019										
	**Unhealthy foods**		**Tobacco**		**Alcohol**		**Gambling**		**Weight loss**		**Other policies related to content targeted at minors**
	General	*Minors*	*General*	*Minors*	*General*	*Minors*	*General*	*Minors*	*General*	*Minors*	
											make any deceptive and/or unsubstantiated claims. All claims and assertions need to be substantiated within the video itself.<br>(ii) Paid Ads cannot imply that the product will improve your social status.
WhatsApp	**NO**	**NO**	**YES**<br><br>**-** Businesses may not transact in the sale of tobacco products or tobacco paraphernalia		**YES**<br><br>**-** Businesses may not transact in the sale of alcohol		**YES**<br><br>- Businesses may not transact in or facilitate gambling, games of skill, or lotteries, including online casinos, sports books, bingo, or poker if it costs money		**YES**<br><br>**-** Businesses may not transact in deceptive, false, misleading, or offensive offers or products. **Diet, weight loss, or other health related products that imply or attempt to generate negative self-perception		
Facebook Messenger	**NO**	**NO**	**YES - Prohibited Content**<br><br>- Adverts **must not promote**		**YES - Restricted Content**<br><br>**-** Ads that **promote or reference alcohol**	**YES - Restricted Content**<br><br>***Targeting requirements:***	**YES - Restricted Content**<br><br>- Adverts that **promote or facilitate online**	**YES - Restricted Content**<br><br>- Authorised gambling,	**YES - Prohibited Content**<br><br>**-** Ads must not contain "before-	**YES - Restricted Content**<br><br>- Ads marketed	

Platform	Advertising provisions as at December 2019										
	Unhealthy foods		Tobacco		Alcohol		Gambling		Weight loss		Other policies related to content targeted at minors
	General	*Minors*	*General*	*Minors*	*General*	*Minors*	*General*	*Minors*	*General*	*Minors*	
			**the sale or use of tobacco products** and related paraphernalia		**must comply with all applicable local laws, required or established industry codes, guidelines, licences and approvals, and include age and country targeting criteria** consistent with Facebook's targeting guidelines and applicable local laws.<br>- Note that ads promoting or referencing alcohol are prohibited in some countries, including, but not limited to: Afghanistan, Brunei, Bangladesh, Egypt, Gambia, Kuwait, Libya, Lithuania, Norway, Pakistan, Russia, Saudi Arabia, Turkey, United Arab Emirates and Yemen	If you choose to run adverts that promote or reference alcohol, you must make sure that you follow local laws and target your adverts appropriately, including targeting your adverts to people aged 18 years or older in all permitted countries, except the following:<br>***India***:<br>- 25 years or older in Chandigarh, Delhi, Haryana, Maharashtra and Punjab<br>- 21 years or older in Andhra Pradesh, Arunachal Pradesh, Assam, Chhattisgarh, Dadra and Nagar Haveli, Dama and Diu, Goa, Jammu and Kashmir, Jharkhand, Kerala, Madhya Pradesh, Meghalaya,	**real money gambling, real money games of skill or real money lotteries, including online real money casinos, sport books, bingo or poker, are only allowed with prior written permission**. - Authorised gambling, games of skill or lottery adverts **must target people over the age of 18 who are in jurisdictions for which permission has been granted**	games of skill or lottery adverts **must target people over the age of 18 who are in jurisdictions for which permission has been granted**	and-after" images or images that contain unexpected or unlikely results. Ad content must not imply or attempt to generate negative self-perception in order to promote diet, weight loss or other health-related products.	at or for weight loss must be targeted to people aged 18 years or older.	

Platform	Advertising provisions as at December 2019										
	**Unhealthy foods**		**Tobacco**		**Alcohol**		**Gambling**		**Weight loss**		**Other policies related to content targeted at minors**
	General	***Minors***	***General***	***Minors***	***General***	***Minors***	***General***	***Minors***	***General***	***Minors***	
						Odisha, Tamil Nadu, Telengana, Tripura, Uttar Pradesh, Uttarakhand or West Bengal<br>- 18 years or older in the Andaman and Nicobar Islands, Himachal Pradesh, Karnataka, Mizoram, Puducherry, Rajasthan and Sikkim.<br>- Alcohol is prohibited in Bihar, Gujarat, Lakshadweep, Manipur and Nagaland.<br><br>***Other countries:***<br>- 20 years or older in Japan, Iceland, Thailand and Paraguay<br>- 21 years or older in Cameroon, Micronesia, Palau, Solomon Islands, Sri Lanka and the US<br>- 19 years or older in Canada,					

Platform	Advertising provisions as at December 2019										
	**Unhealthy foods**		**Tobacco**		**Alcohol**		**Gambling**		**Weight loss**		**Other policies related to content targeted at minors**
	General	*Minors*	*General*	*Minors*	*General*	*Minors*	*General*	*Minors*	*General*	*Minors*	
						Korea and Nicaragua<br>- 25 years or older in Sweden					
WeChat	NO	NO	**Yes - Prohibited**<br><br>- [Translated from Mandarin] Including tobacco products, such as cigarettes, cigarette packs, cigratte casing		NO	NO	**Yes - Prohibited**<br><br>- [Translated from Mandarin] Including gambling machines, products, games of skills, and cheating machines		NO	NO	
Instagram	NO	NO	**YES - Prohibited Content**<br><br>- Adverts must not promote the sale or use of tobacco products and related paraphernalia- Tobacco or tobacco-related products, including cigarettes, cigars, chewing tobacco, tobacco pipes, hookahs, hookah lounges, rolling papers, vaporized delivery devices, and		**YES - Restricted Content**<br><br>- Ads that **promote or reference alcohol must comply with all applicable local laws, required or established industry codes, guidelines, licences and approvals, and include age and country targeting criteria** consistent with Facebook's targeting guidelines and applicable local laws.<br>- Note that ads promoting or	**YES - Restricted Content**<br><br>***Targeting requirements:*** If you choose to run adverts that promote or reference alcohol, you must make sure that you follow local laws and target your adverts appropriately, including targeting your adverts to people aged 18 years or older in all permitted countries, except the following: ***India***	**YES - Restricted Content-**<br><br>Adverts that **promote or facilitate online real money gambling, real money games of skill or real money lotteries, including online real money casinos, sport books, bingo or poker, are only allowed with prior written permission**.<br>- Authorised gambling, games of skill or lottery adverts **must target people over the age of 18 who are in**	**YES - Restricted Content**<br><br>- Authorised gambling, games of skill or lottery adverts **must target people over the age of 18 who are in jurisdictions for which permission has been granted**	**YES - Prohibited Content**<br><br>- Ads must not contain "before-and-after" images or images that contain unexpected or unlikely results. Ad content must not imply or attempt to generate negative self-perception in order to promote diet, weight loss or other health-related products.	**YES - Restricted Content**<br><br>- Ads marketed at or for weight loss must be targeted to people aged 18 years or older.	

Platform	Advertising provisions as at December 2019										
	**Unhealthy foods**		**Tobacco**		**Alcohol**		**Gambling**		**Weight loss**		**Other policies related to content targeted at minors**
	General	*Minors*	*General*	*Minors*	*General*	*Minors*	*General*	*Minors*	*General*	*Minors*	
			electronic cigarettes.		referencing alcohol are prohibited in some countries, including, but not limited to: Afghanistan, Brunei, Bangladesh, Egypt, Gambia, Kuwait, Libya, Lithuania, Norway, Pakistan, Russia, Saudi Arabia, Turkey, United Arab Emirates and Yemen	- 25 years or older in Chandigarh, Delhi, Haryana, Maharashtra and Punjab<br>- 21 years or older in Andhra Pradesh, Arunachal Pradesh, Assam, Chhattisgarh, Dadra and Nagar Haveli, Dama and Diu, Goa, Jammu and Kashmir, Jharkhand, Kerala, Madhya Pradesh, Meghalaya, Odisha, Tamil Nadu, Telengana, Tripura, Uttar Pradesh, Uttarakhand or West Bengal.<br>- 18 years or older in the Andaman and Nicobar Islands, Himachal Pradesh, Karnataka, Mizoram, Puducherry, Rajasthan and Sikkim.<br>- Alcohol is prohibited in	**jurisdictions for which permission has been granted**				

Platform	Advertising provisions as at December 2019										
	**Unhealthy foods**		**Tobacco**		**Alcohol**		**Gambling**		**Weight loss**		**Other policies related to content targeted at minors**
	General	***Minors***	***General***	***Minors***	***General***	***Minors***	***General***	***Minors***	***General***	***Minors***	
						Bihar, Gujarat, Lakshadweep, Manipur and Nagaland.<br><br>***Other countries***<br>- 20 years or older in Japan, Iceland, Thailand and Paraguay<br>- 21 years or older in Cameroon, Micronesia, Palau, Solomon Islands, Sri Lanka and the US<br>- 19 years or older in Canada, Korea and Nicaragua<br>- 25 years or older in Sweden					
QQ	**Not Available**	**Not Available**	**Not Available**	**Not Available**	**Not Available**	**Not Available**	**Not Available**	**Not Available**	**Not Available**	**Not Available**	**Not Available**
Qzone	**Not Available**	**Not Available**	**Not Available**	**Not Available**	**Not Available**	**Not Available**	**Not Available**	**Not Available**	**Not Available**	**Not Available**	**Not Available**
Douyin / Tik Tok	**NO**	**NO**	**NO**	**NO**	**NO**	**NO**	**YES - Restrictions**<br><br>**-** Use, display, upload, modify, publish, transmit, update, share or store any Ad Materials on the Tik Tok Ads Platform which: (i) is harmful, harassing,	**YES - Restrictions**<br><br>**-** Use, display, upload, modify, publish, transmit, update, share or store any Ad Materials on the Tik	**NO**	**NO**	**YES - Restrictions**<br><br>**-** Use, display, upload, modify, publish, transmit, update, share or store any Ad Materials on the Tik Tok Ads Platform which:

Platform	Advertising provisions as at December 2019										
	**Unhealthy foods**		**Tobacco**		**Alcohol**		**Gambling**		**Weight loss**		**Other policies related to content targeted at minors**
	General	***Minors***	***General***	***Minors***	***General***	***Minors***	***General***	***Minors***	***General***	***Minors***	
							blasphemous, defamatory, obscene, obscene, pornographic, paedophilic, libellous, invasive of another's privacy, hateful, or racially, ethnically or otherwise objectionable, disparaging, relating or **encouraging** money laundering or **gambling**, or otherwise unlawful in any manner whatsoever; (ii) **harms minors in any manner**; or (iii) deceives or misleads the addressee of the origin of such messages or communicates any information which is grossly offensive or menacing in nature	Tok Ads Platform which: (i) is harmful, harassing, blasphemous, defamatory, obscene, obscene, pornographic, paedophilic, libellous, invasive of another's privacy, hateful, or racially, ethnically or otherwise objectionable, disparaging, relating or **encouraging** money laundering or **gambling**, or otherwise unlawful in any manner whatsoever; (ii) **harms minors in any manner**; or (iii) deceives or misleads the addressee of the origin of such messages or communicates any			(i) is harmful, harassing, blasphemous, defamatory, obscene, obscene, pornographic, paedophilic, libellous, invasive of another's privacy, hateful, or racially, ethnically or otherwise objectionable, disparaging, relating or **encouraging** money laundering or **gambling**, or otherwise unlawful in any manner whatsoever; (ii) **harms minors in any manner**; or (iii) deceives or misleads the addressee of the origin of such messages or communicates any information which is grossly

**Platform**	**Advertising provisions as at December 2019**										
	**Unhealthy foods**		**Tobacco**		**Alcohol**		**Gambling**		**Weight loss**		**Other policies related to content targeted at minors**
	General	*Minors*	*General*	*Minors*	*General*	*Minors*	*General*	*Minors*	*General*	*Minors*	
								information which is grossly offensive or menacing in nature			offensive or menacing in nature
Sina Weibo	**Not Available**	**Not Available**	**Not Available**	**Not Available**	**Not Available**	**Not Available**	**Not Available**	**Not Available**	**Not Available**	**Not Available**	**Not Available**
Reddit	**NO**	**NO**	**YES - Prohibited Advertisements (Hazardous Products or Services)**<br><br>**-** Advertisers may not use the Platform to promote the use or sale of hazardous, dangerous, or injurious products or services, including products subject to consumer recalls, explosive materials or fireworks, recreational drugs or substances, weapons, guns, ammunition, explosives, **tobacco products**, and		**YES - Restricted Advertisements**<br><br>**-** All alcohol advertisements must be manually approved and certified by Reddit. In order to be approved, the advertiser must be actively working with a Reddit Sales Representative. Additionally, advertisements related to alcoholic beverages or products must: (i) comply with applicable laws and regulations, which may include those applicable to the location of your business and of your target audience;	**YES - Restricted Advertisements**<br><br>- All alcohol advertisements must be manually approved and certified by Reddit. In order to be approved, the advertiser must be actively working with a Reddit Sales Representative. Additionally, advertisements related to alcoholic beverages or products must: (iii) target individuals above the legal-drinking age.	**YES - Restricted Advertisements**<br><br>**-** All gambling advertisements must be manually approved and certified by Reddit. In order to be approved, the advertiser must be actively working with a Reddit Sales Representative.<br>- All approved gambling advertisers must comply with applicable laws, regulations, and licensing requirements, which may include those applicable to the location of your business, those applicable to the location of your target audience, and those related		**NO**	**NO**	**NO**

Platform	Advertising provisions as at December 2019										
	Unhealthy foods		Tobacco		Alcohol		Gambling		Weight loss		Other policies related to content targeted at minors
	General	*Minors*	*General*	*Minors*	*General*	*Minors*	*General*	*Minors*	*General*	*Minors*	
			related products or services.		(ii) comply with industry standards, as may be reflected in the Distilled Spirits Council Code of Responsible Practices, the Beer Institute Advertising and Marketing Code and Guidelines, or the Wine Institute Code of Advertising Standards; and (iii) target individuals above the legal-drinking age.		to responsible gambling.<br>- They must also comply with industry standards, as may be reflected in the American Gaming Association's Code of Conduct for Responsible Gaming (or country equivalent) and ensure minors are not the target of such advertisements.<br><br>The following products and services will be affected by this policy:<br>- Online casinos and gambling where real money is exchanged<br>- Sports betting<br>- Fantasy sports games where real money (or other items of value) is exchanged<br>- Lotteries<br>- Brick and mortar casinos<br>- Games/apps played for money or other items of				

Platform	Advertising provisions as at December 2019										
	**Unhealthy foods**		**Tobacco**		**Alcohol**		**Gambling**		**Weight loss**		**Other policies related to content targeted at minors**
	General	*Minors*	*General*	*Minors*	*General*	*Minors*	*General*	*Minors*	*General*	*Minors*	
							value, such as gift cards				
Twitter	NO	NO	**YES - Prohibited Content**<br><br>-Twitter prohibits the promotion of tobacco products, accessories, and brands globally	**YES - Prohibited Content for Minors**<br><br>- Prohibits knowingly marketing or advertising tobacco products and related accessories, including electronic cigarettes, to minors	**YES - Restricted Content**<br><br>- Twitter restricts the promotion of online and offline sale of alcohol and general awareness of alcohol brands.<br>- These restrictions are based on the specific product or service being promoted, as well as the country that the campaign is targeting.	**YES - Prohibited Content for Minors**<br><br>- Prohibits knowingly marketing or advertising alcoholic beverages and related accessories to minors	**YES - Restricted Content**<br><br>- Twitter prohibits the promotion of gambling content, except for campaigns targeting specified countries where it is allowed with restrictions	**YES - Prohibited Content for Minors**<br><br>- Prohibits knowingly marketing or advertising gambling products and services, including lotteries, to minors	**YES - Restricted Content**<br><br>- Twitter restricts the promotion of health and pharmaceutical products and services. These restrictions are based on the specific product or service being promoted, as well as the country that the campaign is targeting.<br>- This policy applies, but is not limited to: Nutrition products, including weight loss products, marketed with unreasonable or unsubstantiated claims		
Douban	Not Available	Not Available	Not Available	Not Available	Not Available	Not Available	Not Available	Not Available	Not Available	Not Available	Not Available
Snapchat	**YES - Industry-Specific Requirements (Pharmaceutical and Healthcare**		**YES - Prohibited Content (Inappropriate Content**		**YES - Industry-Specific Requirements**	**YES - Restricted Content**<br><br>Ads that promote or	**YES - Industry-Specific Requirements**		NO	NO	

Platform	Advertising provisions as at December 2019										
	**Unhealthy foods**		**Tobacco**		**Alcohol**		**Gambling**		**Weight loss**		**Other policies related to content targeted at minors**
	General	***Minors***	***General***	***Minors***	***General***	***Minors***	***General***	***Minors***	***General***	***Minors***	
	**- Diet and Fitness)**<br><br>- Ads for food products must accurately describe the qualities and characteristics of a food product, including any associated health and nutritional claims		**-** Ads that promote cigarettes (including e-cigarettes), cigars, vaping products, tobacco, nicotine, or related products of any kind		Ads that promote or reference alcohol must not:<br>- Target or be likely to appeal particularly to people under the legal drinking age in the territory where the ad will run.<br>- Encourage or depict excessive or irresponsible consumption of alcohol.<br>- Exploit an individual who is drunk or otherwise intoxicated.<br>- Glamorize alcohol, or otherwise misrepresent the effects of consuming alcohol.<br>- Associate alcohol with operating a vehicle or other activities that require a certain degree of skill or physical coordination, or with any illegal behavior.	reference alcohol must not:<br>- Target or be likely to appeal particularly to people under the legal drinking age in the territory where the ad will run.<br><br>Ads for alcohol products must be age targeted to at least 18+, or the applicable minimum drinking age in the country to which you are advertising:<br>Canada: 19+<br>Japan, Thailand: 20+<br>United States: 21+<br>Sweden: 25+<br>India: either 18+, 21+, or 25+ depending on the States or Union Territories being targeted.	- Our ad policies regarding the promotion of Gambling Services apply to the promotion of online casinos, brick and mortar casinos, lotteries, daily fantasy sports, and any product or service that asks Snapchatters to pay to play games of chance to win prizes with real-world value ("Gambling Services"). Advertisers for Gambling Services must comply with all applicable licensing or registration requirements and must provide Snap with proof of current license or registration. All Gambling Services advertisers must be pre-approved by Snap.				

Platform	Advertising provisions as at December 2019										
	**Unhealthy foods**		**Tobacco**		**Alcohol**		**Gambling**		**Weight loss**		**Other policies related to content targeted at minors**
	General	*Minors*	*General*	*Minors*	*General*	*Minors*	*General*	*Minors*	*General*	*Minors*	
					Mandatory warning labels, such as "please drink responsibly" or its local variation (if applicable) must be prominently displayed in the ad.<br><br>Snap does not permit targeting ads that promote or reference alcohol to the following countries: Algeria, Bahrain, Egypt, Indonesia, Jordan, Kazakhstan, Kuwait, Lithuania, Morocco, Nigeria, Norway, Oman, Pakistan, Qatar, Saudi Arabia, Tunisia, Turkey, and the United Arab Emirates.<br><br>**Ads for alcohol products must be age targeted to at least 18+, or the applicable minimum drinking age in**						

Platform	Advertising provisions as at December 2019										
	Unhealthy foods		Tobacco		Alcohol		Gambling		Weight loss		Other policies related to content targeted at minors
	General	*Minors*	*General*	*Minors*	*General*	*Minors*	*General*	*Minors*	*General*	*Minors*	
					**the country** to which you are advertising:<br>Canada: 19+<br>Japan, Thailand: 20+<br>United States: 21+<br>Sweden: 25+<br>India: either 18+, 21+, or 25+ depending on the States or Union Territories being targeted.						
LinkedIn	NO	NO	**YES - Prohibited Content**<br><br>- Ads for tobacco products are prohibited. This includes ads selling or promoting, directly or indirectly, any tobacco products (such as cigarettes, pipes, cigars), alternative tobacco products (such as e-cigarettes or vaporizers), or any related equipment that facilitates the use of tobacco.		**YES - Restricted Content**<br><br>- Ads for alcoholic products are restricted and only available in certain countries<br>- Contact ad sales team for ads placement		**YES - Prohibited Content**<br><br>**-** Ads related to gambling or sweepstakes of any kind are prohibited		**YES - Prohibited Content**<br><br>- Ads promoting unrealistic or misleading claims about health improvements, including related to **diet and weight loss**, are prohibited.<br>- LinkedIn reserves the right to restrict advertising related to any health matter if targeted inappropriately		

Platform	Advertising provisions as at December 2019										
	**Unhealthy foods**		**Tobacco**		**Alcohol**		**Gambling**		**Weight loss**		**Other policies related to content targeted at minors**
	General	*Minors*	*General*	*Minors*	*General*	*Minors*	*General*	*Minors*	*General*	*Minors*	
			Lawful products and services which promote quitting tobacco are permitted								
Pinterest	**NO**	**NO**	**YES - Prohibited Content**<br><br>Pinterest doesn't allow the advertising of tobacco products including but not limited to:<br>- Cigarettes<br>- E-cigarettes<br>- Chewing tobacco<br>- Paraphernalia (i.e, pipes, rolling papers, vapes, vape cartridges, etc.)<br><br>Pinterest also does not allow the advertising of hookah or cigar bars or products.		**YES - Restricted Content**<br><br>We allow advertising of alcohol, including beer, wine, spirits, liqueurs, and alcohol memberships. In all cases, you're responsible for complying with applicable federal and local laws and regulatory guidelines, including age gating where necessary.<br><br>Alcohol ads on Pinterest:<br>- Can't target minors, or depict minors consuming alcoholic beverages<br>- Can't associate the consumption of alcohol to enhanced	**YES - Restricted Content**<br><br>We allow advertising of alcohol, including beer, wine, spirits, liqueurs, and alcohol memberships. In all cases, you're responsible for complying with applicable federal and local laws and regulatory guidelines, including age gating where necessary.<br><br>Alcohol ads on Pinterest:<br>- Can't target minors, or depict minors consuming alcoholic beverages	**YES - Restricted Content**<br><br>Pinterest doesn't allow the advertising of:<br>- Any kind of lottery<br>- Gambling game apps<br>- Gambling websites<br><br>Pinterest also doesn't allow advertising for fantasy sports or other online prize-based games that require payment for entry. Ads for brick and mortar casinos that don't lead to online gambling, lotteries or games of skill are OK except where prohibited in our country-specific guidelines.		**YES - Restricted Content** (Healthcare products and services)<br><br>Pinterest doesn't allow the advertising of:<br>- Weight loss or appetite suppressant pills and supplements<br>- Unlikely or dangerous weight-loss claims and before-and-after weight-loss imagery		

Platform	Advertising provisions as at December 2019										
	Unhealthy foods		Tobacco		Alcohol		Gambling		Weight loss		Other policies related to content targeted at minors
	General	*Minors*	*General*	*Minors*	*General*	*Minors*	*General*	*Minors*	*General*	*Minors*	
					physical performance, improved social standing or better sexual success<br>- Can't imply that drinking excessively is beneficial, or that alcohol has therapeutic qualities either as a stimulant or relaxant<br>- Can't depict people under the influence of alcohol, or associate drinking with activities that are risky (such as driving, operating heavy machinery), antisocial or illegal<br>- Can't emphasize high alcoholic content as a positive quality						

Table S2: Links to Advertising Policies of Selected Social Media Platforms, as at December 2019

**Platform**	**Link to advertising policy (as at December 2019)**
Facebook	https://www.facebook.com/policies/ads/
YouTube	https://support.google.com/youtube/answer/188570?hl=en-GB&ref_topic=30084&visit_id=637218312152286372-2351322132&rd=1
YouTube Kids	https://support.google.com/youtube/answer/6168681?hl=en
WhatsApp	https://www.whatsapp.com/legal/commerce-policy/
Facebook Messenger	https://www.facebook.com/policies/ads/
WeChat (in Mandarin)	https://ad.weixin.qq.com/guide/77
Instagram	https://www.facebook.com/help/publisher/221149188908254
QQ	Not Available
Qzone	Not Available
Douyin / TikTok	https://ads.tiktok.com/i18n/official/policy/disclaimer
Sina Weibo	Not Available
Reddit	https://www.reddithelp.com/en/categories/advertising/ad-review/reddit-advertising-policy
Twitter	https://business.twitter.com/en/help/ads-policies/introduction-to-twitter-ads/twitter-ads-policies.html
Douban	Not Available
Snapchat	https://www.snap.com/en-US/ad-policies
LinkedIn	https://www.linkedin.com/legal/ads-policy
Pinterest	https://policy.pinterest.com/en/advertising-guidelines

Table S3: Advertising policies of selected multinational children's entertainment networks (Nickelodeon and Disney) in relation to unhealthy food, tobacco, alcohol, gambling and weight-loss, as at December 2019

**Platform**	**Advertising provisions as at December 2019**										
	**Unhealthy foods**		**Tobacco**		**Alcohol**		**Gambling**		**Weight loss**		**Other policies related to content targeted at minors**
	General	*Minors*	*General*	*Minors*	*General*	*Minors*	*General*	*Minors*	*General*	*Minors*	
Nickelodeon [1]	**YES**<br><br>Guiding principles for advertising on Nickelodeon:<br>- **Not promote an unhealthy or inactive lifestyle** and **not encourage excessive consumption of HFSS foods**		**YES**<br><br>The service **must not broadcast a tobacco advertisement** within the meaning of the Tobacco Advertising Prohibition Act 1992 (Cth) (s 10(1)(a) Schedule 2 BSA)		**YES**<br><br>AANA Code of Marketing & Communication to Children (Australia): **Not be for, or relate in any way to, alcohol products or draw any association with companies** that supply alcohol products	**YES**<br><br>AANA Code of Marketing & Communication to Children (Australia): **Not be for, or relate in any way to, alcohol products or draw any association with companies** that supply alcohol products	**YES**<br><br>The Service **must not broadcast an interactive gambling advertisement** within the meaning of the Interactive Gambling Act 2001 (Cth) – relevant sections of this Act are set out in attachment 2 to this Schedule 1		**YES**<br><br>Compliance with other Codes: Under Clause 6 of the Code, advertisements communicated by Licensees (and thus Viacom channels) must broadly comply with:<br>- Weight Management Code of Practice		
Disney [2]	**YES**<br><br>All campaigns for food products must adhere to The Walt Disney Company Nutrition Guidelines**For caffeinated beverages - Ad content reviewed on a case-by-case basis-**	**YES**<br><br>All campaigns for food products must adhere to The Walt Disney Company Nutrition Guidelines**For caffeinated beverages - Ad content reviewed on a case-by-case basis-**	**YES**<br><br>**Inappropriate ad content for all Disney websites**- Tobacco (cigarettes, cigars, pipes, chewing tobacco, etc. and except for anti-smoking campaigns approved by DI)		**YES**<br><br>**Inappropriate ad content for all Disney websites**- Liquor of any kind (hard, beer, wine) (except on Family Network, where only hard liquor is inappropriate)	**YES**<br><br>**Inappropriate ad content for all Disney websites**- Liquor of any kind (hard, beer, wine) (except on Family Network, where only hard liquor is inappropriate)	**YES**<br><br>**Inappropriate ad content for all Disney websites**- Gambling (excluding legal state lotteries, sweepstakes and fantasy leagues)		**YES**<br><br>Ad content reviewed on a case-by-case basis:- Prescription drugs, vitamins, dietary supplements, and diet/weight-loss products		

Platform	Advertising provisions as at December 2019										
	**Unhealthy foods**		**Tobacco**		**Alcohol**		**Gambling**		**Weight loss**		**Other policies related to content targeted at minors**
	General	*Minors*	*General*	*Minors*	*General*	*Minors*	*General*	*Minors*	*General*	*Minors*	
	Caffeinated beverages (products like coffee, which are clearly adult-targeted, are often allowed, while sodas and energy drinks are not)	Caffeinated beverages (products like coffee, which are clearly adult-targeted, are often allowed, while sodas and energy drinks are not)									

Notes:
[1] Nickelodeon's advertising policy is available at http://www.nick.com.au/gsp.scenic/international/nickelodeon.com.au/docs/MTVN_BALA-v2-Nickelodeon_Standards_&_Practices_Australia_and_New.pdf
[2] Disney's advertising policy is available at https://mediakit.go.com/disney/disney-ad-guidelines/
